# The Mutation Responsible for Torsion Dystonia Type 1 Shows the Ability To Stimulate Intracellular Aggregation of Mutant Huntingtin

**DOI:** 10.34763/devperiodmed.20182201.3338

**Published:** 2018-04-12

**Authors:** Marta Jurek, Ewa Obersztyn, Michał Milewski

**Affiliations:** 1 Department of Medical Genetics, Institute of Mother and Child, Warsaw, Poland

**Keywords:** torsin 1A, TOR1A, torsion dystonia, chaperone proteins, protein aggregation, huntingtin, torsyna 1A, TOR1A, dystonia torsyjna, białka opiekuńcze, agregacja białek, huntingtyna

## Abstract

**Introduction:**

Torsion dystonia type 1 is the most common form of early-onset primary dystonia. Previous reports have suggested that torsin 1A, a protein mutated in this disease, might function as a chaperone that prevents the toxic aggregation of misfolded polypeptides.

**The aim of the study:**

The aim of this study was to verify the chaperone function of torsin 1A by investigating its ability to prevent the aggregation of huntingtin model peptides.

**Materials and methods:**

N-terminal mutant huntingtin fragments of different length were co-expressed in neuronal HT-22 and non-neuronal HeLa cells with either the wild-type or mutant (ΔE302/303) torsin 1A protein. The transfected cells were immunostained and analyzed for the presence of huntingtin aggregates using fluorescence microscopy.

**Results:**

The immunofluorescence analysis of huntingtin subcellular distribution within the transfected cells showed no significant difference between the huntingtin aggregation levels in cells co-expressing the wild-type torsin 1A and in control cells co-transfected with an empty vector. Instead, it was the increased level of huntingtin aggregation in the presence of the torsion dystonia-causing ΔE302/303 mutant that reached statistical significance in both neuronal and non-neuronal cells.

**Conclusions:**

Either torsin 1A does not function as a chaperone protein or huntingtin is not an efficient substrate for such a hypothetical chaperone activity. However, the ability of mutant torsin 1A to stimulate the accumulation of aggregation-prone polypeptides might constitute an important source of ΔE302/303 pathogenicity and thus a potential target for future therapy.

## Streszczenie

**Wstęp:**

Dystonia torsyjna typu 1 jest najczęstszą postacią pierwotnej dystonii o wczesnym początku. Kilka wcześniejszych doniesień sugerowało, że torsyna 1A, białko zmutowane w tej chorobie, może pełnić funkcję białka opiekuńczego, zapobiegającego toksycznej agregacji nieprawidłowo zwiniętych polipeptydów.

**Cel badania:**

Celem niniejszej pracy było zweryfikowanie hipotezy o opiekuńczej funkcji torsyny 1A poprzez zbadanie zdolności tego białka do zapobiegania agregacji modelowych peptydów huntingtynowych.

**Materiały i metody:**

N-końcowe fragmenty zmutowanej huntingtyny o różnej długości były koeksprymowane z prawidłową lub zmutowaną (ΔE302/303) torsyną 1A w neuronalnych komórkach HT-22 i nieneuronalnych komórkach HeLa. Transfekowane komórki były poddawane immunobarwieniu i analizowane na obecność agregatów huntingtynowych przy użyciu mikroskopii fluorescencyjnej.

**Wyniki:**

Immunofluorescencyjna analiza subkomórkowej lokalizacji huntingtyny w transfekowanych komórkach wykazała brak znaczących różnic między poziomem agregacji huntingtyny w komórkach eksprymujących prawidłową torsynę 1A i w komórkach kontrolnych transfekowanych pustym wektorem, wskazując na brak znaczącej aktywności opiekuńczej torsyny 1A w badanych warunkach. Statystycznie znaczący okazał się natomiast wzrost poziomu agregacji huntingtyny w obecności patogennego wariantu ΔE302/303 torsyny 1 A, co dotyczyło zarówno komórek neuronalnych, jak i nieneuronalnych.

**Wnioski:**

Otrzymane wyniki wskazują, że torsyna 1A nie pełni funkcji białka opiekuńczego albo też huntingtyna nie jest wydajnym substratem dla owej hipotetycznej funkcji tego białka, jednakże zaobserwowana zdolność mutacji ΔE302/303 w torsynie 1A do stymulowania procesu akumulacji agregujących peptydów może stanowić istotne źródło jej patogenności, a tym samym potencjalny cel przyszłej terapii dla dystonii torsyjnej typu 1.

## Introduction

Dystonias are a group of neurological conditions in which excessive muscle contractions lead to repetitive movements and abnormal postures. It is believed that nearly half of all dystonia cases are secondary or acquired, while the other half includes the so-called idiopathic or primary dystonias. Many forms of dystonia are inherited and there are about 25 distinct chromosomal loci and 16 genes associated with dystonia that are known to date [1]. The most common form of primary dystonia of early onset is torsion dystonia type 1 (DYT1). It is inherited as an autosomal dominant trait and typically presents in childhood or adolescence [2].

DYT1 shows incomplete penetrance, as only 30-40% of all the individuals carrying the pathogenic mutation show any symptoms of the disease, while both the severity and the actual spectrum of clinical features may vary considerably even within the same family. The most characteristic features of DYT1 include involuntary and sustained muscle contractions, with the first symptoms usually affecting a leg or an arm, before developing into a generalized or multifocal dystonia, although in many cases the symptoms remain limited to writer’s cramp only.

Certain medical approaches, like injection of botulinum neurotoxins or deep-brain stimulation, can be used to control the disease symptoms. However, there is currently no effective cure for DYT1. Since this is at least partially caused by our limited knowledge of pathomechanisms responsible for the disease, intensive studies on both the molecular and cellular mechanisms involved in the pathogenesis of DYT1 are needed to develop more effective therapeutical strategies.

Nearly all known cases of DYT1 are caused by the same genetic mutation, which is a deletion of three consecutive nucleotides (c.934-936delGAG) in the *DYT1/TOR1A* gene. On a protein level, this pathogenic mutation results in a deletion of a single glutamic acid residue at position 302/303 (ΔE302/303) in a polypeptide called torsin 1A (TOR1A), an AAA+ ATPase located in the endoplasmic reticulum and the nuclear envelope [2]. Previous studies have shown that TOR1A is important for maintaining the proper structure and function of the nuclear envelope [3, 4, 5, 6] and for regulating the functioning of synaptic terminals [7, 8, 9]. However, a number of reports have also suggested that TOR1A may function as a chaperone protein that suppresses the aggregation of misfolded proteins [10, 11, 12], although it remains unclear whether this potential function of TOR1A is indeed crucial for the pathogenesis of DYT1.

Among different human polypeptides prone to intracellular aggregation, the N-terminal fragments of mutant huntingtin, a protein responsible for Huntington’s disease, constitute one of the most extensively studied models for pathogenic protein aggregation [13, 14]. To investigate whether the over-expression of TOR1A affects the aggregation of mutant huntingtin, we decided to examine the impact of both the wild-type and DYT1-causing TOR1A variant on the intracellular aggregation of different N-terminal fragments of huntingtin containing the pathogenically elongated polyglutamine tract.

## The aim of the study

The specific aim of this study was to verify the hypothetical chaperone function for torsin 1A by investigating its ability to suppress the intracellular aggregation of mutant huntingtin-derived peptides, one of the most commonly studied models for pathogenic protein aggregation. In a broader sense, the aim of this study was to identify a function of TOR1A that plays a crucial role in the pathogenesis of torsion dystonia type 1 and thus may help us elucidate its pathophysiology and eventually develop an effective treatment.

## Material and methods

Neuronal HT-22 cells, derived from a mouse hippocampus, were a gift from K. Domańska-Janik (Mossakowski Medical Research Centre, Warsaw, Poland). The human HeLa cell line was purchased from Sigma-Aldrich (Cat. No 93021013). Both cell lines were cultured under standard cell culturing conditions (5% CO^2^-balanced air at 37^o^C) in DMEM (Gibco) supplemented with 10% fetal bovine serum (Gibco). The construction of the huntingtin-expressing plasmids was described elsewhere [14]. Mammalian expression plasmids encoding both variants of the GFP-TOR1A fusion protein (WT and ΔE302/303) were a gift from X. O. Breakefield (Harvard Medical School, Boston, USA)[15].

ExGen500 (Fermentas) and FuGENE (Promega) transfection reagents were used for the transient transfection of HT-22 and HeLa cells, respectively. Cells were seeded onto collagen-coated glass coverslips and grown for 20-30 hours until reaching 40-60% confluence. In all the cotransfection experiments, a 1:1 DNA ratio was used for plasmids encoding different polypeptides. At 24 hrs after transfection, the cells were fixed in 4% paraformaldehyde for 20 min. at room temperature and immunostained with anti-c-Myc mouse monoclonal antibodies (Sigma-Aldrich) to visualize the c-Myc-tagged huntingtin fragments. The presence of huntingtin aggregates in at least 100 transfected cells was examined in duplicate in two separate experiments using the IX71 fluorescence microscope (Olympus). The standard t-Student test was used to estimate the statistical significance of the differences observed (p<0.01). Images were prepared for publication using the Cell-F (Olympus) and Microsoft Office software.

## Results

**Fig. 1:**
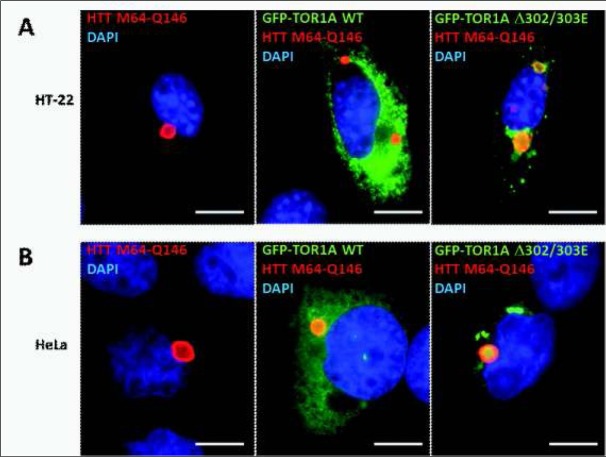
Fluorescence microscopy analysis of cells co-expressing different variants of TOR1A and mutant huntingtin. Exemplary HT-22 (A) and HeLa (B) cells expressing wild-type (WT) or mutated (ΔE302/303) torsin 1A fused with GFP (GFP-TOR1A) and the c-Myc-tagged N-terminal 64 amino-acid fragment of huntingtin containing the pathogenically elongated polyQ tract (HTT M64-Q146). Since huntingtin was stained red with anti-c-Myc antibodies, huntingtin aggregates are seen as red inclusion bodies, usually located in the perinuclear region. The green signal corresponds to GFP-TOR1A, with WT torsin 1A showing mostly reticular staining associated with endoplasmic reticulum, while the mutant protein (ΔE302/303) accumulates in intracellular bodies that are apparently distinct from huntingtin aggregates. The nuclei were stained blue with DAPI. Scale bar - 10 μm. Ryc. 1. Mikroskopowa analiza fluorescencyjna komórek koeksprymujących różne warianty białka TOR1A i zmutowanej huntingtyny. Przykładowe komórki HT-22 (A) i HeLa (B) eksprymujące prawidłową (WT) lub zmutowaną (ΔE302/303) torsynę 1A w fuzji z GFP (GFP-TOR1A) oraz N-końcowy fragment huntingtyny o długości 64 aminokwasów wyznakowany epitopem c-Myc i zawierający patogennie wydłużony ciąg poli-Q (HTT M64-Q146). Ponieważ huntingtynę wybarwiono na czerwono przy użyciu przeciwciał anty-c-Myc, agregaty huntingtynowe widoczne są jako czerwone ciała inkluzyjne, zazwyczaj zlokalizowane w pobliżu jądra komórkowego. Sygnał zielony odpowiada białku fuzyjnemu GFP-TOR1A, przy czym prawidłowa torsyna 1A (WT) wykazuje retikularny wzór lokalizacji charakterystyczny dla siateczki śródplazmatycznej, natomiast zmutowany wariant białka (ΔE302/303) akumuluje się w wewnątrzkomórkowych skupiskach, odmiennych od agregatów huntingtynowych. Jądra komórkowe wybarwione zostały na niebiesko przy użyciu DAPI. Skala liniowa − 10 μm.

**Fig. 2:**
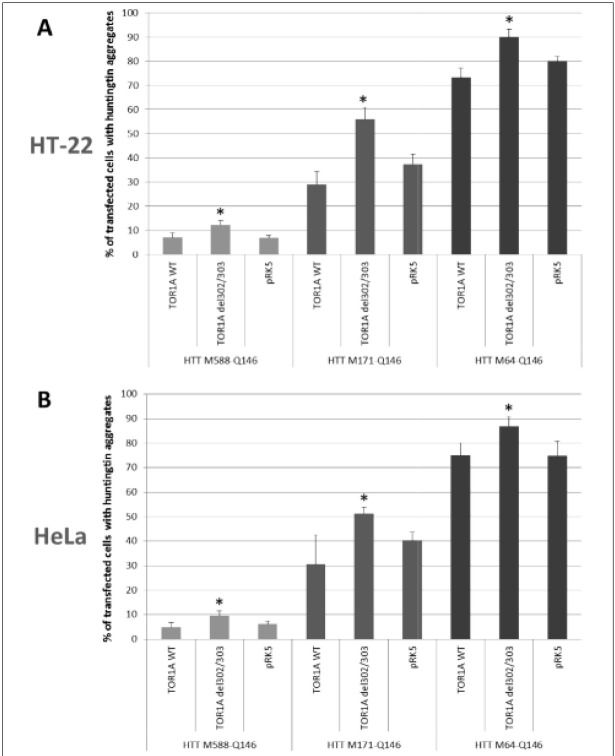
Percentage of transfected cells showing huntingtin aggregation in the presence of over-expressed torsin 1A. The diagrams show the level of huntingtin aggregation in HT-22 (A) or HeLa (B) cells co-transfected with plasmids encoding mutant huntingtin N-terminal fragments of different length (588, 171 or 64 amino acids of the reference sequence) and either torsin 1A (wild-type or mutated protein) or an empty vector (pRK5). Asterisks indicate statistically significant differences in relation to cells over-expressing the same variant of huntingtin in the absence of torsin 1A. Ryc. 2. Procentowy udział transfekowanych komórek wykazujących agregację huntingtyny w obecności nadeksprymowanej torsyny 1A. Diagramy przedstawiają poziom agregacji huntingtyny w komórkach HT-22 (A) lub HeLa (B) kotransfekowanych plazmidem kodującym N-końcowy fragment zmutowanej huntingtyny o określonej długości (588, 171 lub 64 aminokwasy sekwencji referencyjnej) oraz plazmid kodujący torsynę 1A (prawidłową lub zmutowaną) lub pusty wektor plazmidowy (pRK5). Gwiazdkami oznaczono wartości różniące się znacząco od wartości odpowiadających komórkom nadeksprymujacym ten sam wariant huntingtyny przy braku obecności torsyny 1A.

To explore whether torsin 1A inhibits the formation of aggregates composed of mutated human huntingtin, we have used a previously developed model for intracellular huntingtin aggregation, in which the N-terminal mutant huntingtin fragments of different length (588, 171 or 64 N-terminal amino-acids of the reference sequence for human huntingtin) show not only different aggregation rates but also slightly different aggregation pathways [14], although each fragment of mutant huntingtin contains the same pathogenically elongated polyglutamine tract (Q146). Each of those huntingtin fragments was co-expressed with either the wild-type or mutant (ΔE302/303) torsin 1A variant in neuronal (HT-22) or non-neuronal (HeLa) cells transfected with an appropriate pair of expression plasmids. In control experiments, the empty vector (pRK5) was used instead of a plasmid encoding torsin 1A. Figure 1 shows some examples of the immunofluorescence analysis used to examine the presence of huntingtin aggregates in co-transfected cells, while Figure 2 includes two diagrams showing the summary of all the results obtained for different pairs of co-expressed proteins in both HT-22 and HeLa cells.

While different huntingtin fragments showed different aggregation rates, in each particular case the aggregation level was significantly lower when huntingtin was coexpressed with the wild-type torsin 1A variant when compared to its mutated counterpart (Fig. 2). However, this was unlikely to have happened as a result of any potential chaperone activity of torsin 1A, as at the same time we were unable to find any statistically significant difference between the huntingtin aggregation rate in the presence of WT TOR1A and in the control experiments in which the plasmid encoding torsin 1A was replaced by an empty vector. By contrast, such a statistically significant difference was observed when comparing the control cells with those expressing the ΔE302/303 torsin 1A mutant, which suggested that it was the presence of a mutant TOR1A variant that significantly enhanced the huntingtin aggregation in both neuronal and non-neuronal cells and for all huntingtin fragments examined.

## Discussion

Although many AAA+ ATPases are known to function as chaperone proteins [16], there are still some doubts whether TOR1A is able to show this kind of activity and whether this is crucial for the pathogenesis of DYT1. For example, although TOR1A has been initially reported to suppress the aggregation of α-synuclein, a protein involved in the pathogenesis of Parkinson’s disease [10], a more recent study has failed to detect any protective effect of torsin 1A in relation to α-synuclein, including both its aggregation and toxicity [17], thus suggesting that many more studies are needed to unequivocally demonstrate that TOR1A is indeed an effective chaperone protein in relation to any specific aggregating peptide.

Since huntingtin is one of the so-called polyglutamine proteins [18, 19], it should be noted that one of the previous studies showed that torsins are able to suppress intracellular aggregation of polyglutamine proteins [11], which is something we have been unable to confirm in the present study. In this context, it is, however, worth noticing that polyglutamine proteins may significantly differ regarding their abilities to form intracellular inclusion bodies [20, 21]. Also, the above-mentioned study was performed using a very different animal model (Caenorhadbditis elegans) and did not include TOR1A itself, but rather its relatively distant C. elegans homologue from the torsin family of proteins.

When discussing the relationship between the DYT1-causing ΔE302/303 TOR1A variant and protein aggregation, it is also worth noting that this particular mutant form of TOR1A shows a unique ability to form a very specific intracellular protein accumulation when over-expressed in vivo (see Fig. 1). However, these ΔE302/303-associated accumulations are very different from the typical inclusion bodies that are observed in the case of mutant huntingtin and other aggregation-prone peptides, as ΔE302/303 TOR1A is actually found in relatively complex structures that not only contain nuclear envelope-derived intracellular membranes [22] but also seem to include the so-called megaRNP granules, likely arrested during the process of their trafficking from the nucleus to the cell periphery [5]. Whether those unusual structures have anything to do with the above-discovered ability of ΔE302/303 TOR1A to enhance protein aggregation remains currently unknown, but is certainly worth further investigation.

Another point worth discussing in the context of the above-described ability of ΔE302/303 TOR1A to increase the aggregation rate of mutant huntingtin is the potential specificity of this phenomenon, especially in relation to all remaining mutant proteins associated with the so-called conformational diseases [23]. This is of course related to the exact mechanism responsible for this kind of protein aggregation enhancement, as it remains unknown whether this is caused by any specific protein-protein interactions involving both torsin 1A and a misfolded or aggregation-prone peptide, or maybe ΔE302/303 TOR1A just interferes with one of the general cell protection systems that either prevent toxic protein aggregation or facilitate the degradation of aggregating proteins. Finding an answer to this question should help us better understand the DYT1 pathophysiology and may also bring us closer to effective therapy.

## Conclusions

1. Torsin 1A is either a relatively ine&cient chaperone protein or its spectrum of aggregation-prone substrates is limited to certain specific peptides and does not include any N-terminal fragments of mutant huntingtin.
2. The presence of the DYT1-causing TOR1A variant ΔE302/303 seems to enhance the pathogenic accumulation of aggregation-prone peptides, which might constitute an important element of the pathomechanism of torsion dystonia type 1 and thus is worth investigating in the context of developing new strategies for disease treatment.
